# Treatment with pioglitazone induced significant, reversible mitral regurgitation

**DOI:** 10.1186/1475-2840-7-12

**Published:** 2008-04-30

**Authors:** Mozhgan Dorkhan, Magnus Dencker, Anders Frid

**Affiliations:** 1 Department of Clinical Sciences, Division of Diabetes & Endocrinology, Lund University, Malmö University Hospital, Malmö, Sweden; 2 Department of Clinical Sciences, Unit of Clinical Physiology and Nuclear Medicine, Lund University, Malmö University Hospital, Malmö, Sweden

## Abstract

There has in recent years been great concern about possible cardiac side effects of thiazolidinediones (TZDs). We present a case-report of a 60 year-old male who developed significant mitral regurgitation during six months treatment with pioglitazone in parallel with laboratory indications of fluid retention. Echocardiography six months after discontinuation of medication showed regression of mitral regurgitation and the laboratory parameters were also normalized. It is noteworthy that six months treatment with pioglitazone could induce significant valve dysfunction, which was reversible, and this underlines the importance of carefully monitoring patients when placing them on treatment with TZDs.

## Introduction

There is an ongoing discussion concerning the possible effects of thiazolidinediones (TZDs) on the heart. In a recent meta/teleo-analysis Singh et al concluded that heart failure might occur at both high and low doses, usually weeks to months after initiating TZDs and in patients without a history of heart failure [1].

## Case presentation

**Figure 1:**
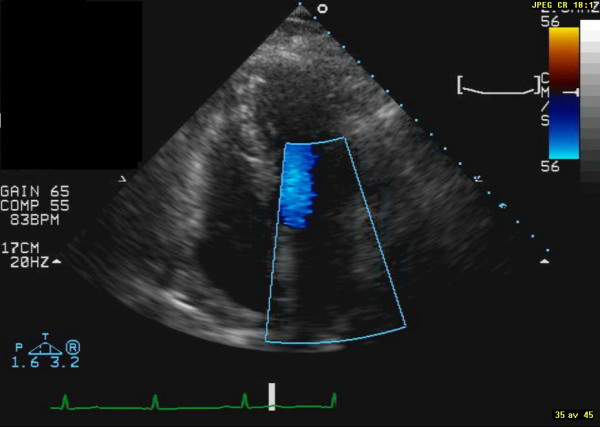
Display of end-systolic image from apical 4-chamber view before treatment with pioglitazone.

**Figure 2:**
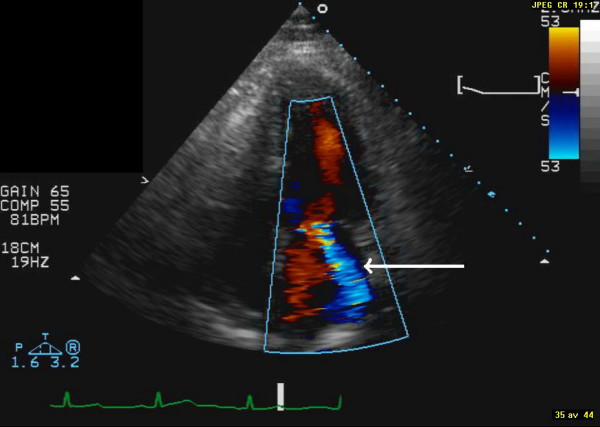
**Display of end-systolic image from apical 4-chamber view after six month of treatment with pioglitazone. **The white, thicker, arrow indicates the newly developed moderate mitral regurgitation.

The patient is a sixty-year-old man with type 2 diabetes known since 15 years. He has proliferative retinopathy and microalbuminuria, no history or clinical signs of cardiovascular disease, stopped smoking 30 years ago. Failure to achieve optimal glycaemic control on treatment with 850 mg metformin/day (reduced dose because of elevated creatinine) and glimepiride 6 mg/day was evident with HbA1c >10%. The patient entered an ongoing study. He received add-on treatment with pioglitazone 30 mg/day that was increased after 16 weeks to 45 mg/day since HbA1c was 8.3% and no side effects were recorded. Standard transthoracic echocardiography examinations were performed with Sonos 5500 (Philips Medical, Best, the Netherlands) at baseline (15/12 2006), after six months pioglitazone therapy (25/6 2007), and six months after discontinuation of pioglitazone (20/12 2007). Cardiac size and volumes were quantified according to current guidelines [2] and echocardiography estimate of left ventricular filling pressure were calculated as previously described [3]. Valvular regurgitation was graded visually (0.5/1/2/3-trivial, mild, moderate, and severe) [4]. Brain BNP and haemoglobin were also measured at start and after six months treatment. After 26 weeks of treatment the HbA1c decreased to 7.4% in parallel with a decrease in haemoglobin from 113 at start to 100 g/l, a weight gain of 3 kg and an increase in brain BNP from 5 to 22 pmol/l. The patient did not show any clinical signs or symptoms of cardiopulmonary affection or peripheral oedema. Echocardiography examination at baseline and at the two subsequent examinations showed a mitral valve with normal morphology, no prolaps or thickened leaflets could be observed. The baseline echocardiography examination showed trivial mitral regurgitation, whereas the echocardiography examination after six months of pioglitazone therapy showed newly developed moderate mitral regurgitation, with a predominantly central jet. Furthermore, ejection fraction was increased and left ventricular- and atrial dimensions were also increased, compared to baseline values. In addition, estimate of left ventricular filling pressure (E/Em) was slightly increased. All the echocardiography alterations and laboratory indications of fluid retention (BNP and haemoglobin) were back to baseline values six months after discontinuation of pioglitazone therapy. Figure 1, 2, 3 display echocardiography images from apical 4-chamber view at baseline, after six month pioglitazone therapy and six months after discontinuation of pioglitazone therapy, respectively. Summary of anthropometrical, echocardiography and laboratory data are displayed in Table 1.

**Table 1:** Descriptive statistics of anthropometrical, echocardiography and laboratory data.

**Date**	**15/12 2006**	**25/62007**	**20/12 2007**
Weight (kg)	82.5	85.7	85.7
HbA1c (%)	10	7.4	7.7
BNP (pmol/l)	5	22	8
Haemoglobin (g/l)	113	100	116
LVDD (mm)	42	49	41
LVSD (mm)	22	24	22
IVS (mm)	10	10	10
Post (mm)	9	9	9
LA (mm)	35	41	35
LV diastolic vol (ml)	100	131	104
LV systolic vol (ml)	34	35	33
EF (%)	66	73	68
LA vol (ml)	53	68	54
E/A	0.7	0.9	0.7
E/Em	10	12	10
Mitral regurgitation (1–3)	0.5	2	1

**Figure 3:**
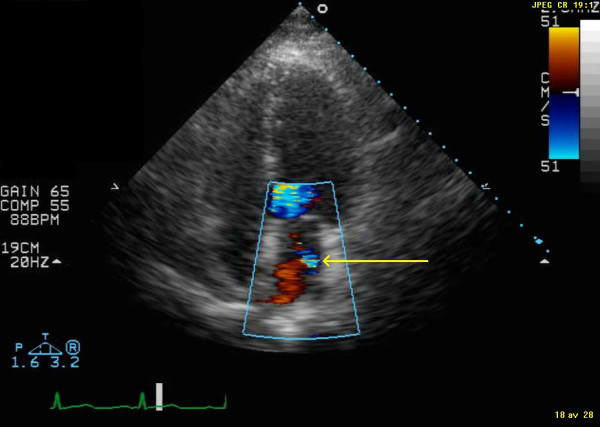
Display of end-systolic image from apical 4-chamber view six months after discontinuation of pioglitazone. The yellow, thinner, arrow indicates the remaining mild mitral regurgitation.

## Discussion

This case-report highlights the importance of careful monitoring of patients under treatment with TZDs. In this case, changes in haemoglobin and BNP indicated fluid retention in the absence of clinical signs or symptoms of cardiopulmonary stress or oedema. What caused the mitral regurgitation? One plausible hypothesis is that the fluid retention caused left ventricular dilatation which in turn resulted in mitral annular dilatation and to altered papillary muscle orientation, which then leads to inadequate mitral leaflet coaptation. Mitral regurgitations that are caused by left ventricular dilatation tend to have a central jet, as in our patient. It is, however, possible that there was some structural weakness in the mitral valve apparatus that was not detected on the transthoracic echocardiography examinations and that this could in part be contributing to the regurgitation. There is a need for tools to help clinicians to identify subsets of patients for whom this kind of therapy is likely to have particularly favourable/unfavourable effect, using readily identifiable clinical and laboratory factors. BNP, being a peptide hormone released from the cardiac ventricles in response to myocyte stretch have generated a lot of attention in recent years and have been proposed as potential diagnostic and prognostic marker for cardiac disease but currently there are no clear algorithms on how this should be implemented into clinical practice. However, the knowledge is increasing in this field and hopefully the results will enable clinicians to select proper therapy for individual patients in future.

## Abbreviations

BNP: Brain natriuretic peptide; LVDD: Left ventricular end-diastolic diameter; LVSD: Left ventricular end-systolic diameter; IVS: End-diastolic inter-ventricular septum; Post: End-diastolic posterior wall; LA: Left atrial end-systolic diameter; LV diastolic: Left ventricular end-diastolic volume; LV systolic vol: Left ventricular end-systolic volume; EF: Ejection fraction; LA vol: Left atrial volume; E/A: Indices of left ventricular diastolic function; E/Em: estimation of left ventricular filling pressure.

## Competing interests

The authors declare that they have no competing interests.

## Authors' contributions

MDo and AF conceived the idea to write this manuscript. MDe performed the echocardiography examinations. All authors wrote, and edited this manuscript together.
